# Neonatal outcomes in twin pregnancies in Finland from 2008 to 2023

**DOI:** 10.1007/s00431-025-05996-y

**Published:** 2025-01-28

**Authors:** Ilari Kuitunen

**Affiliations:** 1 Institute of Clinical Medicine and Department of Pediatrics, University of Eastern Finland, Kuopio, Finland; 2 Department of Pediatrics, Kuopio University Hospital, Puijonlaaksontie 2, 70210 Kuopio, Finland

**Keywords:** Epidemiology, Register study, Perinatal mortality, Neonatal intensive care, Surveillance

## Abstract

Twin pregnancies are associated with higher risks of adverse maternal and neonatal outcomes compared to singleton pregnancies. This retrospective nationwide cohort study analyzed trends in twin pregnancy outcomes in Finland from 2008 to 2023 using data from the Finnish Medical Birth Register. Outcomes assessed included perinatal mortality, stillbirths, neonatal mortality, neonatal intensive care unit (NICU) admissions, and hospitalization rates at one week of age. A total of 23,588 twin births were included, with an overall stillbirth rate of 9.0 per 1000 and a perinatal mortality rate of 16.0 per 1000. Neonatal mortality rates declined significantly, with term twins showing a rate of 0.9 per 1000 and preterm twins 4.6 per 1000 in the latest years of 2022–2023. NICU admission rates remained stable for preterm twins but showed an increasing trend for term twins. The rate of hospitalized neonates at the age of seven days decreased over time.

*Conclusion*: These trends align with improved antenatal care and Finland’s reputation for low neonatal mortality. However, increasing maternal age and obesity rates may contribute to rising NICU admissions in term twins. The study highlights the need for continuous monitoring of neonatal outcomes to ensure high standards of care in the context of declining fertility and delivery rates in Finland.

**Table:** 

**What is Known:** *• Twin pregnancies are associated with higher risks of adverse maternal and neonatal outcomes compared to singleton pregnancies.* *• Finland has one of the lowest neonatal mortality rates globally.*	
**What is New:** *• Neonatal mortality rates declined significantly both in term and preterm twins from 2008 to 2023.* *• NICU admission rates remained stable for preterm twins but showed an increasing trend for term twins.*

**What is Known:**

*• Twin pregnancies are associated with higher risks of adverse maternal and neonatal outcomes compared to singleton pregnancies.*

*• Finland has one of the lowest neonatal mortality rates globally.*

**What is New:**

*• Neonatal mortality rates declined significantly both in term and preterm twins from 2008 to 2023.*

*• NICU admission rates remained stable for preterm twins but showed an increasing trend for term twins.*

## Brief report

Twin pregnancies have a higher risk of adverse maternal and neonatal outcomes than singleton pregnancies [1]. The most recent report from Finland analyzed twin pregnancy outcomes in years 1987–2014. The study found that the perinatal mortality rate had decreased notably from 1987 to 2014 in Finland [2]. All maternal adverse events had increased in twin pregnancies during the study period, which was partly explained by the increasing age and weight of the parturients [3]. As the fertility has been rapidly decreasing in Finland, it is important to continuously monitor the pregnancy outcomes [4]. In Finland, the age of the parturients has been increasing and the body mass index has been rapidly increasing [5]. Thus, the aim of this brief report was to analyze the most recent trends in twin pregnancy neonatal outcomes in Finland.

This was a retrospective nationwide cohort study from 2008 to 2023 based on the open-access database of the Finnish Medical Birth Register. Data was restricted to twin pregnancies. The following outcomes were selected for analysis: perinatal mortality, stillbirths, neonatal mortality, need for neonatal intensive care unit admission, and hospitalization rate at one week of age. Incidences with 95% confidence intervals (CI) were calculated per 1000 deliveries.

**Fig. 1:**
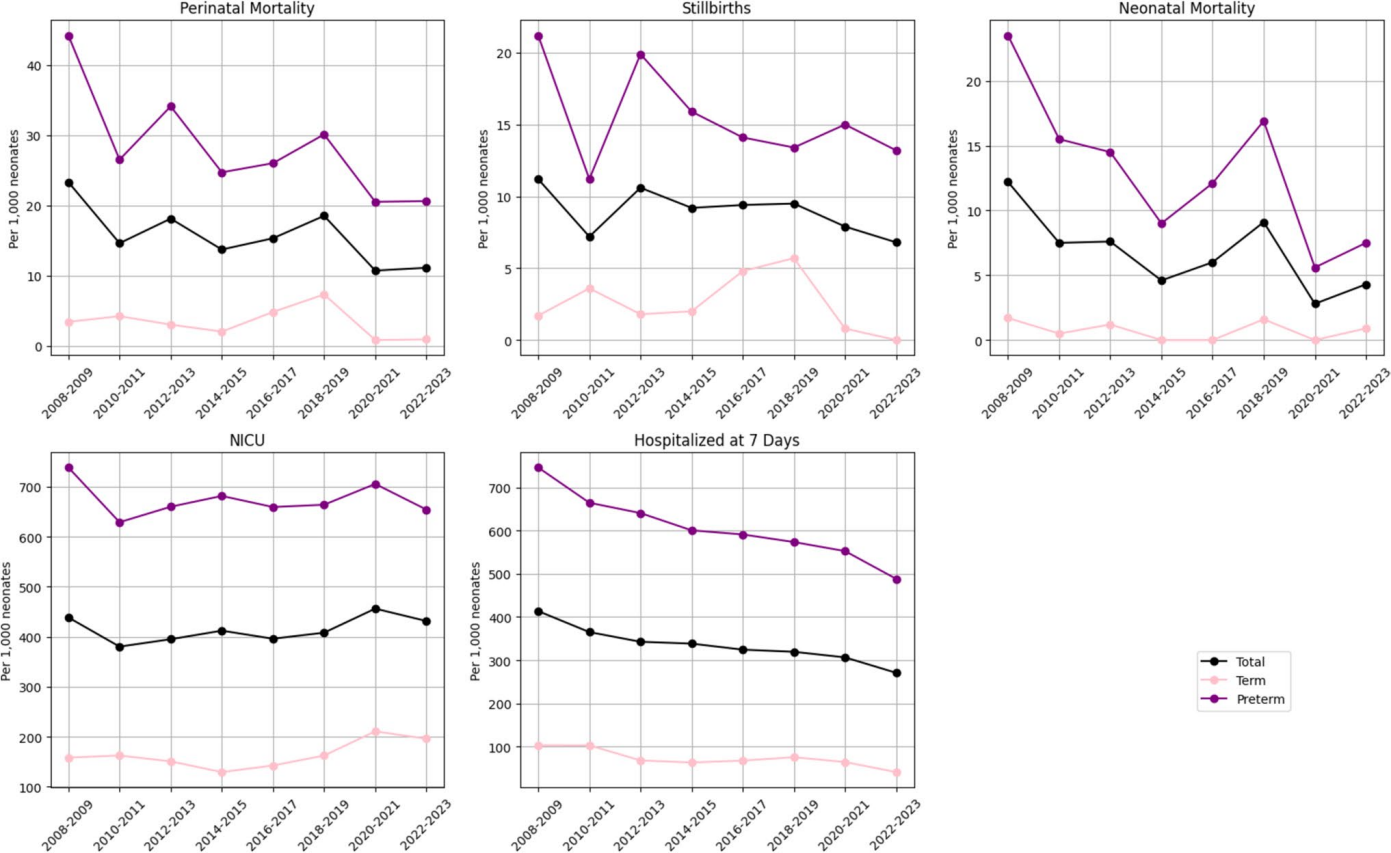
Neonatal outcomes stratified by preterm status and overall, per 1000 born twins from 2008 to 2023

A total of 23,588 twin born were included, and 213 were stillborn. The overall stillbirth rate during the study period was 9.0 per 1000. The overall perinatal mortality rate was 16.0 per 1000, and the rate was notably higher in preterm born twins (Table 1). The neonatal mortality rate has been decreasing (Fig. 1). The overall rate during the study period was 0.8 per 1000 for term and 13.5 for preterm born neonates (Table 1). The lowest reported neonatal mortality rate for term born has been 0.0 per 1000 and 5.6 per 1000 for preterm born neonates in the years 2020–2021 (Fig. 1). The need for neonatal intensive care unit has been rather stable throughout the study period for preterm twins but showed an increasing trend towards the end of the study period in term twins (Fig. 1). The overall rates for term born were 161.8 per 1000 and for preterm 674.6 per 1000 (Table 1). A clear decrease is seen in the hospitalization time, as 745.8 per 1000 preterm twins were hospitalized at seven days in 2008–2009 compared to the latest rate of 488.3 per 1000 (Fig. 1). Similar trend is seen among term born twins.

**Table 1:** Incidences per 1000 deliveries of neonatal outcomes in twin pregnancies from 2008 to 2023

Outcome	N of events	N of population	Incidence (CI)
Perinatal mortality	377	23,558	16.0 (14.4–17.7)
Term	41	11,938	3.4 (2.5–4.6)
Preterm	336	11,620	28.9 (25.9–32.1)
Stillbirths	213	23,558	9.0 (7.9–10.3)
Term	31	11,938	2.6 (1.8–3.6)
Preterm	182	11,620	15.7 (13.5–18.1)
Neonatal mortality	164	23,346	7.0 (6.0–8.2)
Term	10	11,908	0.8 (0.4–1.5)
Preterm	154	11,438	13.5 (11.5–15.7)
NICU admissions	9 643	23,346	413.0 (404.9–421.4)
Term	1927	11,908	161.8 (154.7–169.2)
Preterm	7716	11,438	674.6 (659.7–689.8)
Hospitalized at 7 days	7948	23,346	340.4 (333.0–348.0)
Term	910	11,908	76.4 (71.6–81.5)
Preterm	7038	11,438	615.3 (601.1–629.8)

Overall, the perinatal and neonatal mortality trends have been decreasing in Finland throughout the years. A previous study reported the decreasing trend from 1987 to 2014 [2], and this current study further confirms continued trend until 2023. The latest neonatal mortality rate of 4 per 1000 aligns to similar levels as reported in Japan (4 per 1000), Netherlands (6 per 1000), UK (6 per 1000), and lower than in Australia (18 per 1000), and the USA (22 per 1000) [6–10]. However, except from Australia, these studies provided older results for comparison as the current study. A recent European multinational study reported that Finland, Sweden, and Norway had the overall lowest neonatal mortality rate in Europe [11]. Thus, these findings of the neonatal mortality rate in twin pregnancies are in line with the overall results. As both the stillbirths and neonatal mortality rates were decreasing, this can be seen as an improved overall antenatal care. Although the rates of neonatal intensive care unit admissions seemed to be increasing for term twins and remained stable for preterm twins, the hospitalization time decreasing both in preterm and term born neonates. A contributing factor to the increase in intensive care unit admission is the higher maternal age and obesity rate in Finland, as both of these are associated with increased neonatal morbidity [5]. The previous study from Finland found that the relative gap between the neonatal outcomes of the A and B twin had increased from 1987 to 2014 [2].

Due to the crude nature of this current data set, more specific information on the neonatal outcomes, and especially on the outcome comparison of A and B twins was not available. Alternative quality indicators, such as need for therapeutic hypothermia, umbilical pH measurements, and need for invasive ventilation are needed when analyzing the quality of neonatal care. This information will be available earliest within a year, due to current long waiting times for register-based studies in Finland [12]. The main strength is the excellent coverage and quality of the Medical Birth Register, and the uniform reporting practice in all hospitals.

In conclusion, the decreasing trend for perinatal and neonatal mortality has continued in Finland. It is important to continuously monitor the neonatal outcomes due to rapidly decreasing fertility rates, which lead to lower numbers of overall deliveries but also lower numbers of high-risk deliveries per delivery unit. Future analyses should study whether the previous gap in the outcome of the second born twin has been managed to reduce in Finland.

## Data Availability

All data used is available from the open access database of the Finnish Medical Birth Register: https://thl.fi/en/statistics-and-data/statistics-by-topic/database-reporting#Sexual%20and%20reproductive%20health.
